# Hypersensitivity Reactions Associated with Platinum Antineoplastic Agents: A Systematic Review

**DOI:** 10.1155/2010/207084

**Published:** 2010-09-20

**Authors:** Nektaria Makrilia, Ekaterini Syrigou, Ioannis Kaklamanos, Leonidas Manolopoulos, Muhammad Wasif Saif

**Affiliations:** ^1^Oncology Unit, 3rd Department of Medicine, Sotiria General Hospital, Athens University School of Medicine, Mesogion 152, 115 27 Athens, Greece; ^2^Department of Clinical Oncology, Yale University School of Medicine, New Haven, CT 06510, USA

## Abstract

Platinum-containing chemotherapy agents (cisplatin, carboplatin, oxaliplatin) have been approved in the first-line setting of numerous malignancies, such as ovarian, bladder, head and neck, colorectal, and lung cancer. Their extensive use over the last decade has led to a significant increase in the incidence of hypersensitivity reactions, which are defined as unforeseen reactions whose signs and symptoms cannot be explained by the known toxicity of these drugs. Skin rash, flushing, abdominal cramping, itchy palms, and back pain are common symptoms. Cardiovascular and respiratory complications can prove fatal. Multiple pathogenetic mechanisms have been suggested. Hypersensitivity usually appears after multiple infusions, suggesting type I allergic reactions; however, other types of hypersensitivity also seem to be implicated. Several management options are available to treating physicians: discontinuation of chemotherapy, premedication, prolonging of infusion duration, desensitization protocols, and replacement with a different platinum compound after performing skin tests that rule out cross-reactions among platinum agents.

## 1. Introduction

Platinum-based compounds were first synthesized in the nineteenth century but their clinical use against cancer did not start until the 1970s. Their activity consists of forming DNA addicts that inhibit replication and lead to apoptosis [1, 2]. They have been approved for the treatment of numerous malignancies, such as ovarian, primary peritoneal carcinoma, bladder, head and neck, colorectal, pancreatic, esophageal, gastric, testicular, endometrial, biliary tract, and lung cancer and mesothelioma [3–15]. Platinum agents are also effective in pediatric tumors [16, 17]. They are used in the first-line and adjuvant setting but also as retreatment regimens when there is a long progression-free interval after treating with the same drugs [18, 19].

Cisplatin [(*SP*-4-2)-diamminedichloroplatinum] was the first of the platinum drugs to be used and is characterized by neurotoxicity, nephrotoxicity, ototoxicity, and emetogenicity [1]. Second-generation platinum derivative carboplatin [*cis*-diammine (cyclobutane-1,1-dicarboxylate-*O*, *O*′)platinum(II)] differs from cisplatin in the substitution of two chlorides by a 1,1-cyclobutane dicarboxylate group. Its efficacy in the treatment of many of the above malignancies is equal to that of cisplatin, and its toxicity profile is more favorable [20]. Thus, carboplatin has often been used in place of cisplatin [21, 22]. Oxaliplatin [[(1*R*,2*R*)-cyclohexane-1,2-diamine](ethanedioate-*O*,*O*′)platinum(II)] is a third-generation platinum compound widely used for the treatment of metastatic colorectal cancer and a variety of other malignancies, such as breast cancer, melanoma, non-Hodgkin lymphoma, and head and neck cancer [23–25]. Common adverse events are myelotoxicity, nausea, vomiting diarrhea, paresthesia, and dysesthesias [26–28]. 

Hypersensitivity to a chemotherapeutic agent is defined as an unforeseen reaction whose signs and symptoms cannot be explained by the known toxicity of the drug [29]. Industrial complex platinum salts have been reported to cause hypersensitivity and asthma among refinery workers since 1945 [30]. Hypersensitivity reactions in patients receiving cisplatin were first described in the 1970s in patients who had been retreated with the drug [31]. Since then, all platinum agents have been associated with such reactions.

The aim of this paper is to provide recent data concerning hypersensitivity reactions to platinum-containing chemotherapy agents. We will present the characteristics and pathogenetic mechanisms of this hypersensitivity, as well as the basic management options available to date.

## 2. Incidence

Extensive use of platinum compounds in chemotherapy during the last decade has led to a significant increase in the incidence of hypersensitivity reactions (Table 1). Hypersensitivity to carboplatin is rarely observed during the first course of treatment. In fact, the most striking difference of carboplatin hypersensitivity, when compared to nonplatinum agents, is the fact that most allergic reactions are reported after the patient has received a significant number of infusions although he has exhibited no hypersensitivity up until then [32–37]. Half of the reactions observed are moderate to severe [32]. During the first five cycles, the overall risk is less than 1% [38]; it rises sharply to 6.5% with the sixth cycle [39] and has been reported as high as 27% in patients receiving more than seven cycles of treatment [32, 40, 41]. The overall incidence of carboplatin hypersensitivity reaches 44% in the third-line retreatment setting [32, 42]. Reactions to carboplatin are usually observed after a median number of eight infusions [32, 33, 39]. Markman et al. were the first to report that peak incidence of carboplatin reaction is observed during cycle 2 in the second-line setting [32]. The incidence of cisplatin hypersensitivity exhibits similar characteristics to those observed with carboplatin. It seems to range from 5% to 20% and increases with concomitant radiation [43]. Oxaliplatin-related hypersensitivity reactions were originally considered less common, but with the growing use of this drug during the last years, it has been found that reactions occur at a frequency as high as 18.9% [44, 45]. However, severe grade 3 or 4 events occur in only 1.6% of patients [46]. These reactions usually develop after six drug infusions [47]. Severe anaphylaxis is rarely observed and is mostly described in case reports [48, 49]. 

To the best of our knowledge, there have still been no reports of hypersensitivity reactions associated with newer platinum compounds (i.e., satraplatin, picoplatin). This, however, may be partly attributed to the fact that for some of the trials conducted, known hypersensitivity to platinum-containing compounds was an exclusion criterium. The prevalence of hypersensitivity to these agents remains to be explored in larger studies in the future.

## 3. Risk Factors

Factors predictive of hypersensitivity reactions to platinum agents are yet to be clarified. HLA phenotype and intensity of exposure influence the possibility of sensitization in refinery workers [67]. Prior history of allergy to drugs and various environmental factors are associated with greater risk [68, 69]. 

 In patients receiving carboplatin, the number of prior platinum treatments and total lifetime exposure is associated with possibility of hypersensitivity [32, 34, 69–72], but it is not clear whether lowering individual doses during retreatment would reduce the risk [34]. The interval between the last infusion of first-line chemotherapy and retreatment is an independent predictive variable [69]. High rate of drug infusion also seems to be vital [16] whereas selection of first-line and retreatment regimens is not influential [69]. Most recently, Sugimoto et al. showed that patients with carboplatin-free interval >13 months was related to a 22-fold higher risk of hypersensitivity and that a maximum dose/body of carboplatin >650 mg is also an independent risk factor [40]. Navo et al. observed that carboplatin hypersensitivity is more frequent in patients with ovarian cancer [70]. A retrospective study by Lafay-Cousin et al. in pediatric patients with low-grade glioma concluded that hypersensitivity reactions occurred significantly earlier in children receiving weekly carboplatin infusions as compared to children on a monthly schedule. Being a girl was identified as a risk factor. The cumulative risk of allergic reactions increased with the number of infusions, and there was no evidence of a plateau [17]. It has also been found that the eosinophilic number and ratio during the previous cycle of allergy and on the day of an allergic reaction are not helpful in predicting a hypersensitivity response [73]. 

The incidence of carboplatin hypersensitivity also seems to be associated with the type of antineoplastic drugs it is combined with. More specifically, the CALYPSO trial showed that hypersensitivity reactions occurred more frequently in patients receiving carboplatin-paclitaxel, as compared to patients on a combination of carboplatin with pegylated liposomal doxorubicin (PLD) (18.8% versus 5.6%, resp.) [74]. Markman et al. also reached the conclusion that administering PLD with carboplatin substantially reduces the incidence of carboplatin-associated hypersensitivity [75].

 In oxaliplatin regimens, younger age, female sex, and use of this platinum compound as salvage therapy have recently been recognized as potential risk factors [46] as opposed to type and number of metastatic sites [45].

## 4. Pathophysiology

The exact mechanism of platinum allergy remains unclear (Table 2) although multiple studies have tried to explain the underlying pathophysiology. Reactions are thought to be mainly caused by type I IgE-mediated or type IV T-cell-mediated hypersensitivity. Nevertheless, the presentation pattern of these reactions is unpredictable, suggesting that various immunological and nonimmunological mechanisms are involved [30]. 

 In type I hypersensitivity reactions, IgE immunoglobulin causes the degranulation of mast cells and basophils leading to the nonimmune-mediated histamine and cytokine release [32, 38, 76, 77]. Early-onset symptoms, such as itching, chest pain, rash, and anaphylactic reactions are attributed to this pathway [32]. Hypersensitivity in exposed refinery workers [30, 39], the need of multiple infusions for sensitization, and positive carboplatin skin tests are in favor of the above hypersensitivity mechanism but no study has searched for platinum-specific IgE [30, 78, 79]. Type IV hypersensitivity to platinum agents also seems to be involved. Antigens binding to the major histocompatibility complex activate T cells that have already been sensitized. This hypersensitivity is associated with the delayed inflammatory reaction presenting hours or even days after the infusion [32, 80]. Increased production of cytokines, such as tumor necrosis factor (TNF)-alpha and interleukin (IL)-6, might be caused by platinum compounds acting as superantigens, but this has yet to be proven [80, 81]. According to the latest hypersensitivity models, serotonin is also implicated in anaphylactic mechanisms, and this could explain the symptoms of hypertension and bronchospasm [82].

As far as oxaliplatin is concerned, most reactions seem to be IgE-mediated but type II hypersensitivity is also implicated with the presentation of hemolysis and thrombocytopenia [83]. Chronic urticaria, joint pain, and proteinuria after oxaliplatin regimens have been attributed to type III allergic reactions [73]. Furthermore, idiosyncratic reactions characterized by chills, fever, abdominal cramps, and chest tightness have been described during the infusion [81, 84, 85].

 The development of carboplatin hypersensitivity after a median number of eight therapy courses has been attributed to the fact that the patient is exposed to very low concentrations of “free” platinum during each infusion. The threshold for the manifestation of a reaction is considered to be lowered with each treatment [68, 86]. According to Markman et al., the patient is sensitized during the first-line treatment (6 courses), and retreatment with the same drug provides the additional immunological stimulation necessary for a reaction during infusion number 8 [32]. Novel studies have suggested that hypersensitivity to platinum agents is not only caused by their common platinum component but also by their different structures and biological targets. According to molecular analyses, cisplatin, carboplatin, and oxaliplatin affect various genes, many of which regulate cell cycle and signal transduction [87, 88].

## 5. Symptoms

Platinum hypersensitivity symptoms may develop acutely during infusion or within minutes, hours, or days after the infusion [32]. A mild rash may be the first manifestation of hypersensitivity, and this will be followed by more severe reactions in 50% of patients. Symptoms of mild hypersensitivity include skin rash, urticaria, flushing, palmar itching, burning, edema of the face and hands, abdominal cramping and diarrhea, back pain, and pruritus. They usually resolve quickly with antihistamines and steroids. More severe reactions manifest with bronchospasm, tachycardia, hypotension or hypertension, seizures, and chest pain. Systemic anaphylaxis may be life-threatening [30, 32, 34, 39, 76, 89, 90].

 In oxaliplatin-based regimens, dysesthesia with laryngeal spasm may occur during, immediately after, or even hours after the drug administration [86, 91–93]. Respiratory problems may be more severe than initially estimated in many cases, as hypoxemia in oxaliplatin-related reactions is often found with no symptoms of dyspnoea [73].

## 6. Treatment Strategies

It is recommended that treating physicians inform patients receiving platinum agents of the risks of hypersensitivity, especially after multiple infusions. It is important for patients to be alert and appropriately educated, so that symptoms are promptly recognized. Administration of platinum drugs should take place in a medical setting with all necessary equipment, and the staff should be experienced in treating hypersensitivity reactions. When a reaction occurs, the infusion should be immediately stopped, and normal saline, intravenous antihistamines, and low-dose corticosteroids should be administered (Table 3). The main dilemma is whether the platinum-based regimen should be completely discontinued in future infusions. When the reaction is life-threatening, all platinum compounds are usually excluded from future treatment options. In case of mild-to-moderate hypersensitivity reactions, there is a range of management options [34].

### 6.1. Infusion Rate and Premedication

Lowering of infusion rate and use of premedication with corticosteroids and antagonists of histamine receptors have allowed successful readministration of the platinum agent in some case; however there is great risk of recurrence. Some studies actually suggest that in carboplatin patients who still have not exhibited hypersensitivity after a total of 8 infusions, premedication should be given as precaution, and the administration rate should be significantly decreased in order to lower the future risk [34]. A retrospective study was most recently conducted by O'Ceabhaill et al. and included 777 patients with relapsed ovarian, fallopian tube, or primary peritoneal cancer retreated with carboplatin. The data showed that an incrementally increasing, 3-hour infusion during carboplatin retreatment, along with appropriate premedication, may reduce hypersensitivity reactions, when compared to the standard 30-minute infusion [34].

### 6.2. Desensitization Protocols

Various desensitization protocols have been successfully implemented in order to re-administer the same platinum agent, especially in cases where the patient seems to have been benefited by the drug (Table 4). Protocols are based on gradual reintroduction of small amounts of drug antigen while escalating to the full dose, on prolonged infusion and premedication. Successful desensitization protocols are usually time-consuming [30, 32, 33, 38, 50, 53–57, 89–91]. Prolonged desensitization is probably more tolerable when considering the changes caused in the extracellular fluid drug concentration rate [94]. However, rapid protocols that develop desensitization within 4–8 hours have also been attempted [78]. A recent trial by Castells et al. included 413 cases and reported a 12-step intravenous and intraperitoneal rapid desensitization protocol that can be applied in a wide range of chemotherapy agents (Table 5). The population consisted predominantly of women with ovarian cancer, therefore, its efficacy, and safety remain to be proven in other populations as well [53]. It should be noted that there have also been reports of successful desensitization in children [95]. Risks and benefits of desensitization protocols must be carefully weighed, and patients should be informed of the danger as there is still risk of anaphylaxis, or even death during the platinum rechallenge [89, 90, 96].

### 6.3. Substituting with a Different Platinum Agent

Substituting one platinum agent with another without additional desensitization is another management option available in case of hypersensitivity [32, 97–100]. Successful replacement of carboplatin by cisplatin has been demonstrated in women with gynecological malignancies [57, 101] but the true incidence of cross-reactivity between platinum salts is not yet known. The possibility of developing a reaction to the substituting platinum agent may be as high as 25% [101], and cases of fatal cisplatin reactions after carboplatin hypersensitivity have been reported [89, 102].

### 6.4. Skin Testing

Skin testing has been used in an effort to predict hypersensitivity reactions in patients about to be re-administered platinum salts [30, 103]. Markman et al. reported that skin tests had been positive in six of seven patients who later developed anaphylaxis during carboplatin readministration [103], thus, suggesting that hypersensitivity reactions can be predicted. Zanotti et al. [30] used similar skin testing to identify patients at risk and reported a 99% negative predictive value. It seems that skin tests are positive in more than 80% of reactive patients and that when they are negative, the risk of hypersensitivity reactions is reduced sevenfold or even eliminated [30, 78]. Therefore, it has been recommended that skin testing is performed in every patient before administration of the eighth dose.

Skin testing can prove helpful in an effort to rule out cross-reactions when one platinum salt is substituted by another [100, 104, 105]. More specifically, patients testing negative for cross-reaction in skin tests seem to be able to safely continue chemotherapy with a different platinum compound. Platinum-based treatment is safely administered without need of pretreatment, and this can prove useful in patients with contraindications to prolonged corticosteroid exposure [102]. It should be noted that patients with positive skin tests were also more likely to exhibit hypersensitivity reactions during desensitization protocols as compared to patients with negative skin tests [52].

The usefulness of skin testing in everyday clinical setting remains an issue of controversy. It does not seem practical to use it as a routine screening test in all patients retreated. It requires prior experience and use of a control. Furthermore, hypersensitivity reactions might still occur even in case of negative skin tests. However, many experts support that skin testing can be extremely helpful when the expected prevalence of hypersensitivity is high. There is need for larger studies in the future in order to clarify the role of skin testing and to establish proper guidelines. 

As far as oxaliplatin hypersensitivity is concerned, pretreatment regimens with corticosteroids, antihistamines, and extension of administration time have been somewhat successful but their reliability has not yet been proven [63, 84, 91, 106–108]. Brandi et al. [107] and Sui et al. [109] reintroduced oxaliplatin in patients having received prophylactic Dexamethasone and antihistamines but hypersensitivity recurred in a significant number of patients. Similar data were reported by two Japanese retrospective studies [45, 73]. Oxaliplatin desensitization protocols have been reported in a very small number of patients [15, 56, 58–66, 110] with the total infusion time ranging from 2 to 24 hours (Table 6). Better response rates and longer time-to-progression were observed with chronomodulated (10 am–10 pm) oxaliplatin infusion [111]. Wrzensinski et al. added magnesium sulfate and calcium gluconate to a previously published protocol and suggested that this may increase the success rate of desensitization [60]. Skin testing for oxaliplatin is 75% to 80% accurate according to Meyer et al. [110] and Garufi et al. [112].

## 7. Conclusions

Platinum-induced hypersensitivity reactions are a potentially fatal complication that occurs at a rising incidence rate due to the growing use of these agents in chemotherapy. The clinical symptoms range from a mild rash to severe anaphylaxis, and multiple types of hypersensitivity seem to be implicated. Doctors as well as patients should be appropriately educated to promptly recognize symptoms. In case of severe anaphylactic reactions, platinum-based treatment is usually completely discontinued. In more moderate reactions, patients can be rechallenged with infusion rate reduction and premedication, by following a desensitization protocol or by receiving a different platinum chemotherapy agent. Skin testing can help rule out the possibility of cross-reaction between platinum-based compounds rendering continuation of effective platinum-based chemotherapy safe.

## Figures and Tables

**Table 1:** Incidence and severity of hypersensitivity to platinum agents.

Drug	Overall incidence %	Characteristics/Severity
Cisplatin	5–20	(i) Occurs within minutes of infusion start
		(ii) Mostly between 4th–8th course
		(iii) Increases with concomitant radiation

Carboplatin	1–44	(i) Occurs within minutes or days from infusion
		(ii) less than 1% during cycles 1–5
		(iii) 6.5% in cycle 6
		(iv) 27% in cycle 7 or more
		(v) 44% in 3rd-line retreatment
		Half of all reactions observed are moderate to severe

Oxaliplatin	10–18.9	(i) Occurs within minutes/hours from infusion
		(ii) Mostly after 6th course
		(iii) Grade 3-4 in only 1.6%
		(iv) Severe anaphylaxis mostly in case reports

**Table 2:** Types of hypersensitivity reactions, their characteristics, and how they are implicated in platinum agent hypersensitivity.

Type of hypersensitivity	Antigen	Mediated by	Mechanism	Involved in platinum hypersensitivity	Symptoms related
I	Soluble antigen	IgE	Mast cell and basophil degranulation	Carboplatin, Cisplatin, Oxaliplatin (most)	Early onset symptoms: itching, chest pain, rash, anaphylactic reactions

II	Cell- or matrix- associated antigen	IgG, IgM	Phagocyte and NK-cell activation	Oxaliplatin	Hemolysis, thrombocytopenia

III	Soluble antigen	IgG	Immune complexes, Phagocyte and NK-cell activation, complement fixation	Oxaliplatin	Chronic urticaria, joint pain, proteinuria

IV	Soluble or cell-associated antigen	T-cells (TH1, TH2, Cytotoxic T cells)	Macrophage and eosinophil activation, cytotoxicity	Carboplatin, Cisplatin	Delayed reactions, hours or even days after infusion

**Table 3:** Emergency procedures in case of hypersensitivity reactions.

Emergency procedures in case of hypersensitivity reaction to a platinum agent
(1) Interruption of infusion
(2) Administer: i.v. antihistamines (type 1 and 2 histamine receptor antagonists), normal saline infusion, and low-dose corticosteroids
(antihistamines are the first to be administered)
(3) In case of a more severe reaction (dyspnoea, laryngospasm, bronchospasm):
(i) oxygen, bronchodilators
(ii) high dose steroids (doses range between 100 and 1000 mg of hydrocortisone)
(4) Administer epinephrine in case of hypotension or airway obstruction symptoms
(5) Monitoring until symptoms resolve or for several hours later in case of severe hypersensitivity

**Table 4:** Main desensitization protocols for carboplatin-cisplatin hypersensitivity. We observe that all protocols deliver the platinum agent at escalating concentrations and administer premedication.

	Patients	Premedication dose route	Steps	Duration	Success rate
Abe et al. (2010) [50]	3 (carboplatin replaced by cisplatin after desensitization)	Dexamethasone 20 mg, promethazine 50 mg, ranitidine 50 mg	From 1 : 1000 to 1 : 10 on 1st day (in 3 steps), from 1 : 10 to 1 : 1 on 2nd day (in 2 steps)	2 days (3 hours on 1st day, 9 hours on 2nd day)	100%

Gomez et al. (2009) [51]	7	Dexamethasone at least 20 mg iv 30, clemastine 2 mg iv	From 1 : 1000 to 1 : 1 in 4 steps	2 hours	71%

Hesterberg et al. (2009) [52]	30	Fexofenadine 180 mg po and/or desloratadine 5 mg po BID, Dexamethasone 10 mg po	From 1 : 100 to 1 : 1 (in 8–10 steps)	11 hours	99%

Castells et al. (2008) [53]	63 (60 hypersensitive to carboplatin, 3 to cisplatin)	Diphenhydramine or hydroxyzine 25 mg po or iv, famotidine 20 mg iv or ranitidine 50 mg iv, lorazepam (0.5-1 mg po or iv as needed for anxiety)	From 1 : 100 to 1 : 1 in 12 steps	5.8 hours	Severe reactions in 6% of courses (full target dose with treatment)

Confino-Cohen et al. (2005) [54]	23	Dexamethasone 8–12 mg iv, ondansetron iv	From 1 : 1000 to 1 : 1 in 4 steps	6 hours	86.9%

Lee et al. (2005) [55]	31 (1st desensitization in intensive care unit—subsequent as outpatient)	Diphenhydramine 25 mg iv, famotidine 20 mg iv Lorazepam 1 mg (as needed for anxiety)	From 1 : 100 to 1 : 1 in 12 steps(higher concentrations as outpatient)	5.8 hours (inpatient) 3.8 hours (outpatient)	85% courses without symptoms (full target dose with treatment)

Markman et al. (2004) [56]	5 (desensitization and retreatment with carboplatin or cisplatin)	Zileuton 600 mg po QID (5 days), montelukast sodium 10 mg po QD (5 days), indomethacin 50 mg po TID (1 day), albuterol sulfate 8 mg po BID (1 day), famotidine 20 mg iv, dexamethasone 20 mg iv, diphenhydramine 50 mg iv	From 1 : 1000 to 1 : 1 in 4 steps	90 minutes	80%

Jones et al. (2003) [57]	5 (carboplatin replaced by cisplatin after desensitization)	Dexamethasone 20 mg po (4 days ), diphenhydramine 50 mg po (4 days), ranitidine 50 mg iv, dexamethasone 20 mg iv, ondansetron 8 mg iv	From 1 : 1000 to 1 : 1 in 4 steps	2 hours	60%

Rose et al. (2003) [33]	33	Dexamethasone 20 mg po or iv 6 hours before, Dexamethasone 20 mg iv and diphenhydramine 50 mg iv 30 minutes before	From 1 : 1000 to 1 : 1 in 4 steps	16.5 hours	79%

BID: twice a day, iv: intravenously, po: by mouth, QD: once a day, QID: four times a day, TID: three times a day.

**Table 5:** Castells' 12-step desensitization protocol.

Step	Solution	Rate (mL/h)	Time (in minutes)	Volume infused per step (mL)
1	100-fold dilution of final target concentration	2.0	15	0.50
2		5.0	15	1.25
3		10.0	15	2.50
4		20.0	15	5.00

5	10-fold dilution of final target concentration	5.0	15	1.25
6		10.0	15	2.50
7		20.0	15	5.00
8		40.0	15	10.00

9	Concentration was calculated by subtracting the cumulative dose administered in steps 1–8 from the total target dose	10.0	15	2.50
10		20.0	15	5.00
11		40.0	15	10.00
12		75.0	prolonged to complete target dose	232.50

**Table 6:** Published desensitization protocols for oxaliplatin hypersensitivity. We observe that almost all protocols deliver the platinum agent at escalating concentrations and administer premedication.

	Patients	Premedication-Dose-Route	Steps	Duration	Success rate
Syrigou et al. (2009) [58]	3	None	From 1 : 100000 to 1 : 1 in 13 steps	8 hours	100%

Castells et al. (2008) [53]	1	Diphenhydramine or hydroxyzine 25 mg po or iv, famotidine 20 mg iv or ranitidine 50 mg iv, lorazepam (0.5–1 mg po or iv as needed for anxiety)	From 1 : 100 to 1 : 1 in 12 steps	5.8 hours	100%

Nozawa et al. (2008) [59]	1	Famotidine 20 mg iv QD, Dexamethasone 20 mg iv QD, diphenhydramine 50 mg po QID, domperidone 10 mg po QID, hydrocortisone 100 mg iv QD, granistron 3 mg iv QD	From 1 : 10000 to 1 : 1 in 5 steps	8 hours	100%

Wrzesinski et al. (2007) [60]	1	Famotidine 20 mg iv BID, Dexamethasone 20 mg iv QD, diphenhydramine 50 mg iv QD, magnesium sulfate 1 g iv BID, calcium gluconate 1 g iv BID, ondasetron 8 mg iv BID	From 1 : 10000 to 1 : 1 in 5 steps	6 hours	100%

Edmondson et al. (2007) [61]	1	None	From 1 : 10000 to 1 : 2 in 12 steps	205 minutes	100%

Herrero et al. (2006)[62]	5	None	From 0.003 to 0.75 mg/min in several steps	5-6 hours	100%

Mis et al. (2005) [63]	1	Dexamethasone 20 mg iv, prednisolone 20 mg po QID, diphenhydramine 50 mg iv, cimetidine 300 mg iv, magnesium sulfate 1 g iv, calcium gluconate 1 g iv, ondasetron 8 mg iv	From 1 : 10000 to 1 : 1 in 5 steps	8 hours	100%

Lim et al. (2004) [64]	1	Diphenhydramine 50 mg po QID and 30 mg iv QD, metoclopramide 9 mg iv QD	Continuous fixed rate	24 hours	100%

Gammon et al. (2004) [65]	1	Dexamethasone iv, hydrocortisone 100 mg iv, diphenhydramine iv	From 1 : 10000 to 1 : 1 in 5 steps	8 hours	100%

Schüll et al. (2001) [66]	1	Dexamethasone iv (high dose), antihistamine iv, ondasetron 8 mg iv	Continuous fixed rate	48 hours	100%

BID: twice a day, iv: intravenously, po: by mouth, QD: once a day, QID: four times a day.
